# Assessment of menopausal symptoms using modified Menopause Rating Scale (MRS) among middle age women in Kuching, Sarawak, Malaysia

**DOI:** 10.1186/1447-056X-9-5

**Published:** 2010-02-22

**Authors:** Syed Alwi Syed Abdul Rahman, Siti Rubiah Zainudin, Verna Lee Kar Mun

**Affiliations:** 1 Department of Family Medicine, Faculty of Medicine and Health Sciences Universiti Malaysia Sarawak, Kuching, Malaysia; 2 Faculty of Resource Science and Technology, Universiti Malaysia Sarawak, Kuching, Malaysia; 3 Department of Family Medicine, International Medical University, Kuala Lumpur, Malaysia

## Abstract

**Background:**

Menopausal symptoms can be assessed by several tools, and can be influenced by various socio-demographic factors.

**Objectives:**

To determine the commonly reported menopausal symptoms among Sarawakian women using a modified Menopause Rating Scale (MRS).

**Methods:**

By using modified MRS questionnaire, 356 Sarawakian women aged 40-65 years were interview to document of 11 symptoms (divided into somatic, psychological and urogenital domain) commonly associated with menopause.

**Results:**

The mean age of menopause was 51.3 years (range 47 - 56 years). The most prevalent symptoms reported were joint and muscular discomfort (80.1%); physical and mental exhaustion (67.1%); and sleeping problems (52.2%). Followed by symptoms of hot flushes and sweating (41.6%); irritability (37.9%); dryness of vagina (37.9%); anxiety (36.5%); depressive mood (32.6%). Other complaints noted were sexual problem (30.9%); bladder problem (13.8%) and heart discomfort (18.3%). Perimenopausal women (n = 141) experienced higher prevalence of somatic and psychological symptoms compared to premenopausal (n = 82) and postmenopausal (n = 133) women. However urogenital symptoms mostly occur in the postmenopausal group of women.

**Conclusions:**

The prevalence of menopausal symptoms using modified MRS in this study correspond to other studies on Asian women however the prevalence of classical menopausal symptoms of hot flushes, sweating was lower compared to studies on Caucasian women.

## Background

Menopause which is defined as complete cessation of menstruation for twelve months or more is a normal physiological change experienced by middle age women. Some of menopausal symptoms experienced by these women can be severe enough to affect their normal daily activities. Unfortunately majority of these women are not aware of the changes brought about by menopause [1-4]. These symptoms are directly resulted from depletion of estrogen level as women approaches menopausal stage and some of these women begin to experiences these menopausal symptoms early in the perimenopausal phase. The common climacteric symptoms experienced by them can be group into: vasomotor, physical, psychological or sexual complaints. It was also noted in some postmenopausal women with long term estrogen deficiency, changes to the cardiovascular or bone which leads to osteoporosis has been established. It is well documented that menopausal symptoms experienced by women affect their quality of life [5-7]. Many published reports shows variations in menopausal symptoms between Asian and Caucasians women, Asian women suffer lesser of somatic and psychological symptoms when compared to their western counterparts [8-11].

Studies shown that perimenopausal and postmenopausal women have more menopausal complaints compared to premenopausal women. They were noted to complain significantly more of vasomotor, sexual and psychological symptoms compared to premenopausal women [12-14].

Various tools or instruments have been designed to measure and assess symptoms during the menopausal transition, among them is Menopause Rating Scale (MRS) which is designed to assess menopause specific health related quantity of life (QoL) to measure the severity of age/menopause-related complaints by rating a profile of symptoms [15,16].

Limited research data were available regarding menopausal symptoms experienced by women in Malaysia. Although menopause related symptoms have been extensively studied in the western countries, not much data were available in Asia especially in South East Asia including Malaysia. All previous studies on menopausal in Malaysia were conducted in Peninsular Malaysia [17-21].

This study was conducted in Kuching, which is the capital city of Sarawak, the largest state in Malaysia and situated in the Island of Borneo.

There is no documented or published data on menopausal study in Sarawak, and this study aimed to document the menopausal-related symptoms among middle age women of Kuching, Sarawak by using modified Menopause Rating Scale.

## Methods

### Subjects and Setting

This is a cross-sectional study. Conducted from the month of January 2007 to November 2007. This study was approved by Universiti Kebangsaaan Malaysia Research and Ethical Committee. The interview was carried out among women ages of 40 to 65 years who visited the government health centers around Kuching, Sarawak, Malaysia. These health centers which provided primary care services are government healthcare centers under the Ministry of Health, Malaysia. The inclusion criteria consisted of women between the ages of 40 to 65 years who had given consent to participate in this study. Pregnant and breast feeding women, women with uncontrolled medical conditions such as hypertension, diabetes mellitus or heart disease, or who were undergoing treatment for cancer, or were in remission, or who had history of drug or alcohol abuse and on hormone replacement therapy were excluded from the study.

### Instrument and data collection

Questionnaires were divided into three sections:

(1) Socio-demographic data of the women, which included: age, race, religion, marital status, educational level, occupation and average household income.

(2) Menopausal status of the women: The menopausal status was classified according to STRAW (Stages of Reproductive Aging Workshop) classification which divided menopause staging into: *Postmenopausal*; no menstrual bleeding in the previous/last 12 months. *Late perimenopause*; had menstruation in the previous/last 2-12 months but not in the previous/last 2 months. *Early perimenopause*; had increasing irregularity of menses without skipping periods (7 days difference from the beginning of a given cycle to the next, experienced after the previously regular cycle and *Premenopause*; minor changes in cycle length particularly decreasing length of the cycle. To aid statistical analysis, the *early *and *late perimenopausal *transition stages were grouped together as *perimenopausal stage *[22,23].

(3) Menopause Rating Scale (MRS) questionnaire were used as a basis for assessing menopausal symptoms in this study, this is a self-administered instrument which has been widely used and validated and have been used in many clinical and epidemiological studies, and in research on the etiology of menopausal symptoms to assess the severity of menopausal symptoms [16].

The MRS is composed of 11 items and was divided into three subscales:

(a) somatic-hot flushes, heart discomfort/palpitation, sleeping problems and muscle and joint problems; (b) psychological-depressive mood, irritability, anxiety and physical and mental exhaustion and (c) urogenital-sexual problems, bladder problems and dryness of the vagina. Each of the eleven symptoms contained a scoring scale from "0" (no complaints) to "4" (very severe symptoms). The questionnaire was first translated into Malay language by a group of experienced health-workers and language experts, and then translated back to English to validate whether the original meaning of the questionnaire was maintained in the translation; a pilot study was done on 60 women to validate the translated MRS questionnaires. The women were asked whether or not they had experienced the 11 menopausal symptoms shown in the MRS in the previous one month (30 days), however it was noted from the pilot study, these women had difficulties in rating the scale themselves, in order to minimize these difficulties, a face-to-face interviewed was done rather than using self-administered respond. Modifications were also made on the grading method of each item due to difficulties faced by the participant in identifying the severity of the symptoms hence the grading was modified to "present" or "absent" of symptoms. Reliability analysis was performed on the modified Menopause Rating Scale questionnaires with Cronbach's alpha of somatic subscale 0.712, psychological subscale 0.743 and urogenital subscale 0.821. Therefore, this study determined the prevalence of menopausal symptoms and not the severity of the symptoms.

All women were interviewed in Malay language. Face-to-face interview were done on all the women by trained health personnel who had undergone training, as this was important to make sure right answer were given, and explanations can be given if the women were in doubt or unclear about the questions asked. At the reception desk during registration, women who fulfill the criteria were invited to participate in this study. Explanations were given and written informed consent was taken from them.

### Statistical analysis

The Statistical Package for the Social Sciences software Version 14.0 (SPSS, Chicago, IL) was used for univariate analyses. The *X² *test was applied to compare the frequencies of the symptoms among the different menopausal status. The level P < 0.05 was considered as the cut-off value for significance.

## Results

Three hundred and fifty six women completed the study. The mean age of respondents in this study was 50.83 ± 6.30 (SD) years. The mean age at menopause was 51.28 ± 2.28 (SD) years with median of 50 years. Among these women, 82 (23.0%) were premenopausal, 141 (39.6%) perimenopausal and 133 (37.4%) postmenopausal. The Iban, Chinese and Bidayuh were the majority ethnic group in this study. Christians made up 178 (50.0%) of the participants, 256 (71.9%) were married and 319 (89.6%) had 11 or less years of schooling. Majority 164 (46.1%) of the participants were housewives and most of the participants 169 (47.5%) had a monthly household income below RM 1000 (US$ 312). (1 USD = RM 3.2). (Table 1)

**Table 1:** Socidemographic data of participants

Sociodemographic Data	Total(n = 356)	%
Age Group(years)		
40 - 44	65	18.3
45 - 49	100	28.1
50 - 54	81	22.7
55 - 59	73	20.5
60 - 65	37	10.4
		
Ethnic Distribution		
Iban	101	28.4
Chinese	80	22.4
Bidayuh	68	19.1
Malay	57	16.0
Melanau	22	6.2
Others(Lumbawang, Kelabit, Kayan, Penan, Punan)	28	7.8
		
Religion		
Christian	178	50.0
Muslim	75	21.1
Buddhist	81	22.6
Others	22	6.3
		
Marital status		
Married	256	71.9
Widow/divorcee	87	24.5
Single	13	3.6
		
Educational level		
No formal education	78	21.9
Primary level	96	27.0
Secondary level	145	40.7
Tertiary level	37	10.4
Occupation		
Housewife	164	46.1
General worker	48	13.4
Semi-professional	107	30.1
Professional	37	10.4
		
Household income (monthly)		
Below RM 1000	169	47.5
RM 1001 - RM 2000	69	19.4
RM 2001 - RM 3000	78	21.9
RM 3001 - RM 4000	24	6.7
RM 4001 - RM 5000	12	3.4
Above RM 5000	4	1.1

Table 2 shows the frequency of menopausal symptoms as assessed by the modified MRS according to most frequent complaints. The three most prevalent menopausal symptoms for all women (n = 356) were: joint and muscular discomfort 285 (80.1%), physical and mental exhaustion 239 (67.1%) and sleeping problems 186 (52.2%). This was followed by symptoms of hot flushes and sweating 148(41.6%), irritability 135 (37.9%), dryness of vagina 135 (37.9%), anxiety 130 (36.5%), depressive mood 116 (32.6%), sexual problem 110 (30.9%), bladder problems 49 (13.8%) and heart discomfort/palpitation 65 (18.3%).

**Table 2:** Frequency of menopausal symptoms among Sarawakian women aged 40-65 years in Kuching, Sarawak assessed by modified MRS

Menopausal symptoms	n = 356	100%
1. Joint and muscular discomfort	285	80.1
2. Physical and mental exhaustion	239	67.1
3. Sleeping problems	186	52.2
4. Hot flushes, sweating	148	41.6
5. Irritability	135	37.9
6. Dryness of vagina	135	37.9
7. Anxiety	130	36.5
8. Dpressive mood	116	32.6
9. Sexual problems	110	30.9
10. Heart discomfort	65	18.3
11. Bladder problems	49	13.8

Most of the somatic and psychological subscale symptoms occur in the perimenopausal group of women compared to pre- and postmenopausal women. However urogenital symptoms occurred most in postmenopausal women.(Table 3).

**Table 3:** Frequency of menopausal symptoms in the participants according to menopausal status among Sarawakian women aged 40-65 using the modified MRS

Subscale (menopausal symptoms)	All (n = 356)	Premenopausal (n = 82)	Perimenopausal (n = 141)	Postmenopausal (n = 133)
Somatic				
1. Hot flushes, sweating	148(41.6) *	29(35.4)	91(64.5) *, †	28(21.1)
2. Heart discomfort	65(18.3)	3(3.7)	40(28.4) *,†	22(16.5)
3. Sleeping problems	186(52.2)	24(29.2)	94(66.7) *,†	68(51.1)
11. Joint and muscular discomfort	285(80.1)	36(43.9)	129(91.4) *	120(90.2) ‡
				
Psychological				
4. Depressive mood	116(32.6)	13(15.9)	67(47.5)	36(27.1) ‡
5. Irritability	135(37.9)	29(35.4)	77(54.6) *,†	29(21.8) †‡
6. Anxiety	130(36.5)	29(35.4)	78(55.4) *,†	23(17.3)
7. Physical and mental exhaustion	239(67.1)	36(43.4)	107(75.9) *	96(72.2) ‡
				
Urogenital				
8. Sexual problems	110(30.9)	17(20.7)	58(41.2) †	35(26.3) ‡
9. Bladder problems	49(13.8)	8(9.7)	14(9.9)	27(20.3) ‡,**
10. Dryness of vagina	135(37.9)	16(19.5)	58(41.2) *	61(45.9) ‡,**

* Significant difference p < 0.05 compared to premenopausal

† Significant difference p < 0.05 compared to postmenopausal.

‡ Significant difference p < 0.05 compared to premenopausal.

** Significant difference p < 0.05 compared to perimenopausal

## Discussion

The mean age at menopause in this study was 51.28 ± 2.28 years. Although this is slightly higher than studies done in Peninsular Malaysia which reported mean age of menopause between 49.4 to 51.1 years and from studies done in Thailand (48.7 years), Singapore (49.1 years) and other studies on Asian and Caucasian women, our findings still falls between the normal range of menopausal age [3,9,10,13,17,18,20,21].

The assessment tool that we used in our study was based on Menopause Rating Scale (MRS) questionnaire. Although in menopausal symptoms studies few assessment tools were available, we used the Menopause Rating Scale (MRS) questionnaires, these questionnaires has been widely used in many epidemiological and clinical research when investigating the menopausal symptoms, These questionnaires has been validated and translated in many languages, although it is a self-administrated questionnaires, it's used were not only meant to assess the menopausal symptoms but also its severity, however, in our study, modification has to be done on the scaling of the original MRS because we noted that the respondents had difficulties in rating the scales, this could be explained by the fact that nearly half of the respondent studied never had formal education or only studied at primary level, and to minimize the reporting error, face to face interviewed were used instead of self administered by the respondents [15,16].

In our study, the classical presentation of menopausal symptoms; hot flushes, sweating and night sweats were noted to be lower (41.6%) when compared to findings from studies done on western women who were reported to be from 45% to 75%. Similar result were also noted in two other studies done in Malaysia by Dhillon et al. (53.0%) and Ismail (57.l0%), however, our finding of low menopausal classical symptoms were shared by studies done in other Asian countries [3,17,18,24-28].

In this study perimenopausal women were noted to experience more of vasomotor symptoms when compared to other menopausal group of women, and this was also statistical significant (Table 3), this can be explained by the fact that in these group of women, estrogen fluctuation during this phase occurs the most, hence they will experienced the most vasomotor symptoms. Our findings were correspond to studies conducted among Australia and other Caucasian women; where as high as 75% of perimenopausal women experienced bothersome vasomotor symptoms at some point of their transitional period [24-30].

From our study, joints and muscular discomfort; physical and mental exhaustions and sleeping problems (Table: 2) which is from the somatic and psychological subscales were experienced most by perimenopausal followed by postmenopausal women and these was also statistical significant differences when compared to premenopausal women. These findings were also noted to be corresponding to studies conducted Asian and Caucasian women [3,7,10,13,18,19,27,28,31,32].

It is interesting to note that in our study, as much as 35% to 45% of premenopausal women also reported similar symptoms (joint and muscular discomfort, physical and mental exhaustion, anxiety, depressing mood, irritability), this could be explained since most of the somatic or psychological symptoms experienced by these middle age women are not exclusively as a result of changes due to menopause alone, it's could also resulted from other physical, psychological or health related problems which is related to aging in these group of women which can represent as menopausal like symptoms [7,13,22].

In urogential subscale (sexual problems, bladder problems and vaginal dryness), from our study the frequency of these symptoms were experienced mainly by postmenopausal group of women and it was also significant statistically when compared to other menopausal status (Table 3) and similar finding were documented from other studies [3,18,25,27,32].

Natural menopause may strongly contribute to sexual changes experienced by these women, however its need to be emphasized that there are numerous factors which contribute to declining sexual activities in middle age women following menopause [18,33-36].

In our study it was noted that somatic and psychological symptoms were experienced mainly by perimenopausal women compared to the postmenopausal or premenopausal women. However, in the urogenital or sexual symptoms, the postmenopausal women were reported to suffer the most compared to the other two groups and similar findings were reported from other studies [2-4,13,18].

There are several limitations of this study. Although attempts were made to ensure that the study population was as representative as possible of the general population of the Kuching, Sarawak, nevertheless it has to be stated owing to the sampling technique used this might not be entirely possible. Another limitation was, as this was a cross sectional study, it does not exclude other confounding effects of the natural aging process that may influence experience of symptoms. Thirdly, this study used modified MRS questionnaire, translated to Malay, so there is the question of accuracy in translation, although this was done by a group of health-workers and language experts. Although MRS is a self reporting questionnaire, in view of substantial number of women studied does not have formal education, in order to include these illiterate women, interviews were used instead. In collecting data, women are asked to provide some retrospective information, such as menopausal symptoms experienced in the preceding one month, last menstruation etc. Hence recall bias is unavoidable, especially for some elderly women. A final limitation of this study is lack of information on regularity of menstruation. Some subjects could have been misclassified into the incorrect menopause status group.

## Conclusion

The study of middle- age women from Kuching, Sarawak between the ages of 40 and 65 years using modified Menopause Rating Scale (MRS), showed that the mean age of menopause was 51.28 ± 2.28 years.

The menopausal symptoms experienced by them were similar to other Asian women but the prevalence classical menopausal symptoms of hot flushes, sweating was lower compared to studies on Caucasian women.

The most common symptoms reported were from the somatic subscale; joint and muscular discomfort, physical and mental exhaustion and sleeping problems.

The perimenopausal women had the most significant somatic complaints compared to postmenopausal and premenopausal women, while postmenopausal women had the most significant urogenital symptoms compared to pre- and perimenopausal women.

## Competing interests

The authors declare that they have no competing interests.

## Authors' contributions

SARSA participated in the design, coordinating and carried out the study and drafted the manuscript.

SRZ performed the statistical analysis and drafted the manuscript.

VLKM participated in the design and coordinating of the study.

All authors read and approved the final manuscript.
